# Population pharmacokinetic modeling of sulfadimethoxine, sulfadiazine and sulfamethoxazole combined to trimethoprim in pigs

**DOI:** 10.1080/01652176.2025.2565351

**Published:** 2025-09-29

**Authors:** Marine Boulanger, Jean-François Taillandier, Jérôme Henri, Mathias Devreese, Siegrid De Baere, Marlène Lacroix, Aude A. Ferran, Alexis Viel

**Affiliations:** a INTHERES, Université de Toulouse, INRAE, ENVT, Toulouse, France; b EMAD unit, ANSES, Fougères Laboratory, Fougères, France; c Laboratory of Pharmacology and Toxicology, Department of Pathobiology, Pharmacology and Zoological Medicine, Faculty of Veterinary Medicine, Ghent University, Merelbeke, Belgium

**Keywords:** Combination, dosage regimen, population pharmacokinetic modeling, pigs

## Abstract

Sulfonamides (S) are old antibiotics combined with trimethoprim (TMP) for synergistic effects against pathogens responsible for a variety of infections in food-producing animals. In growing pigs, the TMP:S ratio is 1:5 based on human TMP/sulfamethoxazole (SMX) dosing which aims to obtain an *in vivo* ratio concentration of 1:19 considered as optimal against human pathogens. However, different sulfonamides with different pharmacokinetic profiles are used in pigs limiting this direct extrapolation from human. The aim was to conduct a PK study in pigs for three commonly used TMP/S combinations and to analyze data using population pharmacokinetic modeling. We found that a 2-compartment structural model fitted best the four drug PK data. TMP has the highest clearance values (0.48 L/h/kg) compared to SMX (0.21 L/h/kg), SDZ (0.12 L/h/kg) and SDMX (0.015 L/h/kg). SDMX has the longest plasma elimination half-life (14.8 h), followed by SDZ (3.7 h), TMP (2.9 h) and SMX (2.2 h). Monte Carlo simulations (*n* = 50,000 pigs) showed that only for 8.8%, 46.8%, and 76.5% of pigs for TMP/SMX, TMP/SDZ and TMP/SDMX, respectively, the free plasma concentration ratio fell within the range of 1:10–1:50 at the marketed doses administered. These results should be further linked to pharmacodynamics to optimize the use of these important antimicrobials drugs in veterinary medicine.

## Introduction

In its latest report, the European Medicine Agency (EMA) ranked penicillins (32.7%), tetracyclines (23.5%) and sulfonamides (9.4%) as the three most sold classes of antibiotics in food-producing animals across Europe (European Medicines Agency 2023). Discovered and characterized in the 1930s, sulfonamides (S) are synthetic bacteriostatic molecules, still used in human medicine, companion animals and food-producing animals. Classified as antifolates drugs, they exert their antimicrobial action by inhibiting the production of key tetrahydrofolate coenzymes. All sulfonamides compete with *p*-aminobenzoic acid (PABA), resulting in the inhibition of the dihydropteroate synthase (Ovung and Bhattacharyya 2021). Their action is very often potentiated with a diaminopyrimidine such as trimethoprim (TMP). The antimicrobial action of trimethoprim is observed a step further in the same metabolic pathway by inhibiting the dihydrofolate reductase, thus preventing the reduction of the dihydrofolic acid (Bushby 1980), although recent work describes a more complex mechanistic pathway of the TMP/S combinations (Minato et al. 2018). It is generally accepted that the inhibition of the same metabolic pathway results in a synergistic and bactericidal effect of the TMP/S combination against Gram-positive and Gram-negative bacteria, protozoa and coccidian (Bushby 1980; Masters et al. 2003). The recent classification of sulfonamides and trimethoprim by the EMA in category D (‘Prudence’) promotes their use as first-line treatments by veterinarians to limit the use of other antibiotics considered as more critical to human health (European Medicine Agency 2020).

Veterinary drugs for growing pigs are usually administered *via* oral medication through food or drinking water (Lekagul et al. 2019), but also as injectable forms. The TMP:S dose ratio found in a great majority of marketed veterinary products is 1:5. This ratio was historically developed in human medicine for the combination of TMP/sulfamethoxazole (SMX) aiming to achieve an *in vivo* free plasma concentration ratio close to 1:19, that is historically considered to be the most synergistic against a wide range of human pathogens (Bushby 1980). This targeted *in vivo* ratio is constant over time in humans as SMX and TMP share approximately the same elimination half-life (T_1/2_) (9–11 h for SMX and 10–12 h for TMP) (Thompson et al. 2019). However, in veterinary medicine, different sulfonamide molecules are used with different physicochemical properties that affect their pharmacokinetics (PK), challenging the direct transposition of the dose ratio administered in human to food-producing animals and questioning the achievement of this *in vivo* 1:19 ratio. For instance, we recently demonstrated that in broilers, the targeted free plasma concentration ratio of 1:19 is observed only in very few individuals (∼5%) of a simulated flock following an oral administration of a 1:5 dose ratio of TMP/SMX or TMP/sulfadiazine (SDZ) (Boulanger et al. 2024). In lambs, the concentration ratio of TMP/S is not constant and varies from 1:13 after an IV administration of TMP/sulfadimethoxine (SDMX) (dose ratio of 1:5) to 1:300 approximately 2 h after the administration (Ferran et al. 2020). There is limited PK data available for these combinations in pigs: Mengelers et al. (Mengelers et al. 1995) showed that following an IV administration of the combination TMP/SDMX (dose ratio of 1:5), the targeted concentration ratio of 1:19 was only achieved for 4 h and continuously decreased to nearly 1:1000 after 24h. For TMP/SDZ, Baert et al. (Baert et al. 2001) showed that the total plasma concentration ratio after an IV administration (dose ratio of 1:5) appeared to vary less over time, as the ratio started at 1:14 immediately after the administration and remained relatively constant and close to 1:19 until the end of their experiment. The evolution of these TMP:S concentration ratios over time can be explained by the difference in T_1/2_ between molecules. The T_1/2_ reported in the literature for pigs range from 12.4 to 16.2 h for SDMX, from 2.3 to 3.1 h for SMX, from 2.8 to 3.5 h for SDZ, and from 1.81 to 5.7 h for TMP (Nouws et al. 1989; Shimoda et al. 1990; Nouws et al. 1991; Mengelers et al. 1995; Baert et al. 2001). However, most studies used only average plasma concentration values to compute these ratios (meaning that the inter-individual variability of the ratios was not explored). In addition, these average concentrations were not corrected for protein binding. It is important to include the protein binding in the analyses because only the free fraction (i.e. not bound to plasma proteins) of the antibiotics will have an effect on bacteria (Wanat 2020). The PK data on the combinations of TMP/S in pigs available today in the literature are mainly from old studies (Shimoda et al. 1989; Nouws et al. 1991; Nielsen and Gyrd-Hansen 1994; Mengelers et al. 1995; Baert et al. 2001) (between the 1980s and 2001), and none of them have addressed inter-individual variability. The use of non-linear mixed effects (NLME) modeling or population modeling (popPK) for PK data allows quantifying the inter-individual variability from the residual variability and exploring the impact of relevant (biological) covariates by analyzing individual PK data rather than pooled or averaged data (Bon et al. 2018). The popPK analysis then allows, thanks to Monte-Carlo simulations, to predict the PK of a treatment at the population level (such as a flock or herd) with the current dosing regimens and, if necessary, to propose dosage adjustments to increase the probability of efficacy of that treatment (Toutain et al. 2017).

The aim of this study was therefore to generate individual pharmacokinetic (PK) data in pigs for three commonly used TMP/S combinations. Sulfadiazine, sulfamethoxazole and sulfadimethoxine were selected because they are antibiotics approved for use in pigs in different European countries and rank among the most commonly used sulfonamides. Data were used to develop a popPK model that better describes the fate of these drugs, including inter-individual variability, and to predict the distribution and evolution of the TMP:S ratio at usual dosing regimens within a pig population. This will provide the necessary information to propose adjustments in the dosing regimens for these TMP/S combinations in pig production.

## Materials and methods

### Animals

These studies were approved by the French Committee on Animal Research and Ethics (APAFIS #40498-2023012513385989 v2 and APAFIS #34432-2021122012043102 v7) and were performed in accordance with the relevant guidelines and regulations. The authors complied with the ARRIVE guidelines. The animal study involved 34 healthy female pigs (Large white x Landrace), aged 7 to 8 weeks at the beginning of the study and provided by either ANSES (Ploufragan, Brittany, France) or INRAE (Saint-Gilles, Brittany, France). Three independent cross-over studies were conducted with 10, 10 and 14 pigs for the combination TMP/SDZ, TMP/SMX, and TMP/SDMX respectively. Animals had access to water *ad libitum* and were fed individually twice a day with antibiotic-free food. Their environment was climate-controlled with a 12-h light:dark cycle and enriched with balls and chewing toys. Animals had one week of acclimatization before being enrolled in the studies. During the first few days of the acclimatization period, animals were housed by two on a slatted floor. Following the insertion of the catheter in the jugular vein, animals were individually housed for the rest of the experiment to prevent catheter removal. Barriers between pens allowed visual, olfactive and tactile (snout to snout) contacts. Pig welfare and health were monitored at least twice daily during all the studies. At the end of the studies, all the animals were anesthetized (ketamine, 10 mg/kg IV route, Imalgene^®^ 1000) followed by euthanasia (Pentobarbital, 140 mg/kg IV route, Euthoxin^®^).

### Drugs and chemical reagents

Diatrim^®^ (Eurovet Animal Health, Bladel, The Netherlands; 200 mg SDZ + 40 mg TMP per mL) and Adjusol^®^ (Virbac, Carros, France; 83.35 mg SDZ + 16.65 mg TMP per mL) containing SDZ and TMP were used for the parenteral (IV and IM) and oral administration, respectively. Lidoprim S^®^ (Prodivet pharmaceutical, sa/nv, Eynatten, Belgium; 200 mg SMX + 40 mg TMP per mL) and T.S SOL^®^ (Dopharma Research, Raamsdonksveer, The Netherlands; 100 mg SMX + 20 mg TMP per mL) containing SMX and TMP were used for IV and oral administration, respectively. Trisulmix^®^ (Dopharma, Saint Herblon, France; 186 mg SDMX + 40 mg TMP per mL) and Biaprim^®^ (Laboratoire Biard, Arques, France; 187 mg SDMX + 40 mg TMP per mL) containing SDMX and TMP were used for the IV and oral administration, respectively. All drugs contained TMP:S in a ratio of 1:5.

Analytical standards of SDMX, SMX, SDZ sodium salt, TMP and the internal standard (IS) TMP-d9 were obtained from Merck Life Science (Hoeilaart, Belgium). Other ISs, such as SDMX-d6, SMX-d4, SDZ-d4 were purchased from Toronto Research Chemicals. Solvents and reagents used for sample preparation (ethylacetate, acetic acid, dipotassium hydrogenphosphate, potassium dihydrogenphosphate, sodium hydroxide) were of p.a. quality (Merck Life Science), whereas solvents used for UPLC-MS/MS analysis (acetic acid (AA), acetonitrile (ACN), methanol) were of ULC/MS quality (Biosolve, Valkenswaard, The Netherlands). Ultrapure water was freshly prepared using a Milli-Q system (Merck Life Science).

### Surgical procedures

After a week of acclimatization period, a double lumen catheter (Ref. LA7FD20, Mila International, USA) was inserted into the jugular vein using an ultrasound-guided procedure (Butterfly IQ+VET) as described by Furbeyre and Labussiere (Furbeyre and Labussiere 2020) to facilitate blood sampling. Briefly, pigs were pre-anesthetized with an intramuscular (IM) mixture of Xylazine (2 mg/kg, Xylasol^®^) and Ketamine (10 mg/kg, Imalgene^®^ 1000) then followed by gas anesthesia (isoflurane (Iso-Vet^®^) induced at 4% and maintained at 2% in oxygen) throughout the procedure. Ketoprofen (3 mg/kg, Ketofen^®^) was also injected (IM) for pain control and the pigs were closely monitored until their recovery. Catheters were evaluated and flushed daily with heparinized physiological serum and a lock solution (30% glucose and heparin (Heparin Choay^®^ 25000UI/5 mL)) was injected when catheters were not used for more than 24–48 h.

### Experimental PK study and blood collection

Twenty-four hours prior to the drug administration, pigs were weighed and randomly allocated to the IV or oral administration groups for the first period of the cross-over design (referred as occasion 1). Then, they were weighted again and switched between groups of administration for the second period (referred as occasion 2, see Table 1). During the two-week cross-over period, animal weights ranged from 28 to 46.8 kg for TMP/SDZ combination, from 28.3 to 37.2 kg for TMP/SMX combination, and from 23.8 to 43.6 kg for TMP/SDMX combination.

**Table 1.** Single dose of TMP/S combinations administered at licensed regimens to pigs by IV, oral and IM routes.

TMP/S combinations	Route of administration	Dose (mg/kg)	Total number of pigs	Number of pigs per occasion
TMP/SDMX	IV	4 + 18.6	14	4 (occ.1) + 2 (occ.2) + 6 (supplementary IV)
	oral	8 + 37.36		4 (occ.1) + 2 (occ.2)
TMP/SDZ	IV	2.5 + 12.5	10	4 (occ.1) + 4 (occ.2) + 2 (supplementary IV)
	oral	5 + 25		4 (occ.1) + 4 (occ.2)
	IM	2.5 + 12.5		8 (occ.3)
TMP/SMX	IV	6 + 30	10	5 (occ.1) + 5 (occ.2)
	oral	6 + 30		5 (occ.1) + 3 (occ.2)

Abbreviations: IV: intravenous; IM: intramuscular; TMP: trimethoprim; SDMX: sulfadimethoxine; SDZ: sulfadiazine; SMX: sulfamethoxazole; occ. = occasion. For the same TMP/S combination, an occasion corresponds to a sequence of cross-over administration, and the same pig is used on different occasions unless otherwise specified (marked as supplementary IV).

For the IV administration, the injection catheter was briefly flushed with physiological saline solution and 0.06 mL/kg of Diatrim^®^ (TMP/SDZ), 0.15 mL/kg of Lidoprim S^®^ (TMP/SMX) or 0.1 mL/kg of Trisulmix^®^ (TMP/SDMX) was slowly injected as a single dose. The corresponding doses in mg/kg are reported in Table 1. After the injection, the catheter was again flushed with physiological saline.

For oral administration, pigs were manually restrained and received 0.3 mL/kg of Adjusol^®^ (TMP/SDZ), 0.6 mL/kg of T.S SOL^®^ (TMP/SMX) and 0.2 mL/kg Biaprim^®^ (TMP/SDMX) (Table 1) through a feeding tube. After a washout period, all groups were reversed: the IV group received the same TMP/S combination by oral administration and the oral group received the same TMP/S combination by IV injection. Pigs were fastened the night before the administration and were fed approximately 15 min after oral administration.

For the TMP/SDZ combination, an additional IM injection was carried out for 8 pigs after a last wash-out period, where pigs received 0.06 mL/kg of Diatrim^®^ (referred as occasion 3, Table 1).

A rich blood sampling collection design allowed blood samples (1–2 mL) to be collected in heparinized tubes at 0 (before administration), 5 min and 15 min for IV only and 0.5, 1, 1.5, 2, 3, 4, 5, 6, 8, 10, 12, 24, 32, 48, 56h after drug administration of TMP/SDZ and TMP/SMX combinations. For the TMP/SDMX combination, additional blood samples were collected at 72, 80, and 96h after drug administration. Samples were then centrifuged within 30 min of collection during 10 min, 3000x g at 5 °C and plasma were stored at ≤-20 °C until drug quantification.

To help develop and improve the models for SDZ and TMP, raw data from the De Smet’s study (De Smet et al. 2017) were included in the PK analysis. Briefly, pigs received either an oral bolus (conventional: 25 mg/kg of SDZ + 5 mg/kg of TMP or under-dosage: 12.5 mg/kg of SDZ + 2.5 mg/kg of TMP) every 12h for 5 days or an IM administration (conventional: 12.5 mg/kg of SDZ + 2.5 mg/kg of TMP or over-dosage: 25 mg/kg of SDZ + 5 mg/kg of TMP) every 24h for 5 days and blood samples were collected for 5 days.

### *In-vivo* protein binding determination

*In vivo* protein binding of sulfonamides and TMP was measured in a small number of pigs enrolled in each PK study (only after IV routes) (Vegas Cómitre et al. 2021). Briefly, 10 µL of a 1 M potassium phosphate buffer (pH = 7.5) were added to 100 µL of fresh plasma collected at the same time points as for the PK studies either in Pall Nanosep Omega 10K (Ref. OD010C34) (*n* = 4 pigs for TMP/SMX and TMP/SDMX) or in Microcon 30 kDa (Ref. MRCF0R030) (*n* = 2 pigs for TMP/SDZ) ultrafiltration devices. The tubes were then centrifuged during 40 min, 4000 rpm at 25 °C, the filter cartridge was removed and tubes were stored at −20 °C until drug assay.

*Total plasma concentration (Conc_pl_tot_) assay*: see section “Drug Concentration Analysis”

*Free plasma concentration (Conc_pl_free_) assay:* Drug assay were performed by transferring 25 µL of the filtrate to an autosampler vial, followed by the addition of 15 µl of the internal standard working solution (labelled S at 25 µg/ml and TMP-d9 at 10 µg/ml), 50 µl of mobile phase A (0.1% AA in water) and 25 µl water/methanol (50/50, v/v). The sample was vortex mixed for 15 s. A 5-µl aliquot was injected on the UPLC-MS/MS system.

The percentage protein binding (PB) was calculated according to the formula:

(eq 1) $$\text{PB }\left(\%\right)\;=\;\left(\left({\text{Conc}}_{\text{pl}\_\text{tot}}-{\text{Conc}}_{\text{pl}\_\text{free}}\right)/{\text{Conc}}_{\text{pl}\_\text{tot}}\right)*\;100$$

### Drug concentration analysis

The analysis of the total drug concentration was carried out by two independent laboratories.

#### Sulfonamides and TMP analysis in laboratory 1

Plasma samples (50 µL) were spiked with 150 µL of an internal standard (IS) mixture containing Sulfamethopyridazine (SMPZ, 10 µg/mL) and isotopically labelled TMP (TMP^13^CD_3_, 0.1 µg/mL), both diluted in methanol (MeOH). The samples were incubated for 5 min at 1400 rpm and 10 °C, followed by centrifugation at 20000 x g and 4 °C for 10 min. The resulting supernatant (50 µL) was diluted with 200 µL of ultrapure water for analysis.

TMP and SDZ plasma concentrations were determined using liquid chromatography coupled with UV and mass spectrometry (LC/UV/MS). Analyses were performed on an Acquity UPLC^®^ system with a photodiode array (PDA) detector and a Xevo^®^ triple quadrupole mass spectrometer (Waters, Milford, MA, USA). Extracted samples (10 µL) were injected onto an Acquity HSS 18SB column (2.1 mm x 100 mm; 1.7 µm, Waters) and separated with a mobile phase consisting of 55% H_2_O and 45% acetonitrile (AcN), acidified with 0.1% formic acid (FA), over a 2-minute run. The UV detection wavelength was set at 270 nm, with retention times of 1.23 for SDZ and 1.70 min for SMPZ. TMP and TMP-^13^CD_3_ were ionised using electrospray in positive mode (ESI+) and quantified in multiple reaction monitoring mode (MRM) mode with transitions of m/z 291.2→230.0 (TMP, collision energy [Ecoll] 26 eV) and **m/z** 295.2→230.0 (TMP-13CD3, Ecoll 26 eV), both with retention times of 1.16 min.

The method was validated with linear calibration curves weighted by 1/X^2^. Calibration ranges were 0.01–10 µg/mL for TMP (three QC levels: 0.03, 0.3 and 3 µg/mL) and 0.1–500 µg/mL for SDZ (four QC levels: 0.3, 3, 30 and 300 µg/mL). Accuracy ranges from 98% to 116% for TMP and from 95% to 112% for SDZ, with intra- and inter-day coefficient of variation (CV) precision below 14%.

SDMX plasma concentration was determined on an Acquity UPLC^®^ with an UV detector. Extracted samples (10 µL) were injected onto an Acquity BEH C18 column (2.1 × 50 mm, 1.7 µm, Waters) and separated using a gradient elution with H_2_O (0.1% FA)/AcN: 10% AcN at 0 min, 60% AcN at 2 min, 10% AcN at 2.1 min, and 10% AcN at 3 min. The detection wavelength was set at 270 nm, with retention times of 1.34 for SPMZ and 1.84 min for SDMX. This method was validated with a calibration range of 0.1–500 µg/mL and three QC levels (3, 30 and 300 µg/mL), using a linear model weighted by 1/X^2^. Accuracy ranged from 96% to 110% with intra- and inter-day CV precision below 10%.

#### Sulfonamides and TMP analysis in laboratory 2

Sample preparation consisted of 100 µL of plasma, mixed with 25 µL of an internal standard working solution (= SDMX-d6, SMX-d4, SDZ-d4 at 25 µg/mL and TMP-d9 at 10 µg/mL), and 100 µL of acetonitrile (as the protein precipitation solvent). The sample was then vortexed at 2500 rpm for 1 min, and subsequently centrifuged at 13000 rpm for 10 min at 4 °C. 1.5 mL ethylacetate was added to the supernatant, and gently mixed on a roller bank for 15 min. Next, the solvent was evaporated under a gentle nitrogen flow at 40 °C. The extract was redissolved in 125 µL of 0.1% AA in Milli-Q water, and transferred to a conical autosampler vial. A 5-µL aliquot was injected on the UPLC-MS/MS system.

The UPLC-MS/MS instrument consisted of a Quattro Premier XE instrument (Waters, Antwerp, Belgium). For chromatographic separation, an Acquity UPLC BEH C18 (2.1 × 50 mm, 1.7 µm) column, in combination with a VanGuard pre-column of the same type (2.1 × 5 mm) was used. Mobile phase A comprised 0.1% (v/v) AA in water and mobile phase B was acetonitrile. A gradient elution program was used: 0 min − 3.0 min, 90%A/10%B, 3.0–5.5 min, to 5.0% A/95.0% B, 5.5–5.7 min, to 90.0% A/10.0% B, 5.7–7.5 min, 10.0% A/90.0. Flow rate was maintained constant at 0.45 mL/min. The sample compartment was cooled at 8 °C, while the UPLC column was maintained at a temperature of 30 °C. The flow was sent by means of a divert valve from 1.1 min to 2.0 min and from 3.0 to 4.3 min, i.e. the time window in which the analytes elute from the UPLC column, to the triple quadrupole mass spectrometer. Quantification of the compounds was done by means of component specific MRM (Multiple Reaction Monitoring) transitions, as shown in Table S1. Quantification was based on ratio of the analyte peak area to the peak area of its corresponding internal standard.

Method validation of the procedure for the analysis of total (free + bound) S and TMP consisted in analyzing spiked blank plasma samples. The matrix-matched calibration curve ranged from 0.004 to 4 µg/mL levels for TMP and from 0.02 to 100 µg/mL for S. The lower limit of quantification (LLOQ) was 4 and 20 ng/mL for TMP and S, respectively.

Accuracy and precision were assessed (*n* = 6) at the LLOQ and at low, medium, and high concentrations, prepared like the calibrator samples. The full validation (within-run and between-run accuracy and precision) was done for total S and TMP in chicken plasma (results not shown). A cross-validation (within-run accuracy and precision) was done in pig plasma. The method for measuring free analyte concentration was fully validated in pig plasma. Results for the calibration range, LLOQ, and detection limit are in Table S2 (total analytes) and Table S3 (free analytes). Accuracy and precision results are in Table S4 (total analytes) and Table S5 (free analytes).

### Population PK modeling

Individual plasma concentration data of SDZ and TMP with all necessary information (e.g. doses, individual bodyweight) from the De Smet study (De Smet et al. 2017) were added to the datasets and analyzed together with the data generated in this study. All plasma concentration data of SDMX, SMX, SDZ and TMP were analyzed using a non-linear mixed effects model (NLME) with Monolix^®^ (Lixoft, version 2024R1). Population PK parameters were estimated and adjusted according to the Stochastic Approximation Expectation-Maximization (SAEM) algorithm. Standard errors were first approximated by linearization and then estimated using bootstrapping (number of runs = 100) as proposed in Monolix^®^. All data were analyzed simultaneously but a structural model was developed for each drug estimating its own structural parameters (see below). For TMP, the same structural model was used across the different PK studies with the different TMP/S combinations. Concentrations between the limit of quantification (LOQ) and the limit of detection (LOD) were interval-censored and concentrations below the LOD were left-censored. Several structural models (i.e. one-, two- and three-compartment) were tested and selected according to their Corrected Bayesian Information Criteria (BICc, the lowest, the best) and graphical evaluation of the data (plots of the population weighted residuals (PWRES), of the individual weighted residuals (IWRES), of the normalized prediction distribution errors (NPDE)). The primary pharmacokinetic parameters estimated for each molecule were the volume of distribution of the central compartment (V1), the volume of distribution of the peripheral compartment (V2), the total body clearance (CL), the inter-compartment clearance (Q), the absorption rate (ka) and the oral and intramuscular bioavailability (F). The secondary parameters were the area under the curve from 0 to infinity (AUC_0-∞_) and the terminal elimination half-life (T_1/2β_) calculated with the classical equations using the micro-constants (Toutain and Bousquet-Mélou 2004a) estimated from the PK models in Monolix^®^. The population parameters were estimated according to a log normal distribution, except for the bioavailabilities (F), which were estimated based on a logit normal distribution allowing to restrict the distribution limit between 0 and 1. The mixed-effects model is a combination of (1) a structural model, used to estimate the fixed effects, which are defined as typical parameters assumed to be similar for all the individuals in the defined population; (2) a statistical/stochastic model, used to estimate the residual (unexplained) variability (e.g. analytical variability, dosing or sampling errors, model misspecification…) (Bon et al. 2018) and the random effects, which are subject-specific variables including the inter-individual variability (IIV) and the inter-occasion variability (IOV) and (3) a covariate model (see below) used to explain some of the IIV and/or IOV (Bon et al. 2018).

To account for the potential variation of the individual parameters over occasions (one occasion in our study corresponds to one of the 2 periods of the cross-over), an inter-occasion variability (IOV) was added to the model. This assumes that the individual parameters (volumes, CL, Q) could vary between the 2 periods of the cross-over but remain constant within the period (Karlsson and Sheiner 1993). In Monolix^®^, when only IOV is implemented for a parameter, the corresponding IIV is implicitly accounted for, as the IOV captures random variability around the fixed effect for each individual across occasions. In this study, pigs were highly homogeneous in breed and age, further limiting inter-individual variability and supporting the decision to model only IOV for these parameters. Equation (2) describes the full model for the individual parameters taking into account the IOV and IIV variabilities:

(Eq2) $$\text{Log}\left(H_{\text{ik}}\right)=\text{log}\left(H_{\text{pop}}\right)+\eta_{\text{Hi}}+\gamma_{\text{Hi}}$$

where H_ik_ is the value of a structural parameter (e.g. volumes, clearances, …) for a given individual i at the occasion k (IV, oral or IM), H_pop_ is the value defined for the population and η_Hi_ and ɣ_Hi_ are the random effects for the IIV and IOV, respectively. Eta for IIV or IOV were kept in the model only if they were significatively different from zero. The IOV was not implemented for the parameter ka as pigs received the oral solution only once, either during the two weeks of cross-over or in the De Smet study.

Covariates are important to underline possible relationships between subject-specific characteristics (e.g. age, breed, sex, bodyweight) or drug-specific characteristics (e.g. drug formulation for TMP) and the estimated popPK parameters to eventually explain some of the IIV and IOV. One biological covariate was implemented as an occasion-varying covariate in this model, namely “bodyweight” (BW). The complete final model is described in Equation (3):

(Eq3) $$\text{Log}\left({\text{H}}_{\text{i}}\right)=\text{log}\left(H_{\text{pop}}\right)+\beta_{\text{BW}}\text{ x log}\left(\text{BW}/31.1\right)+\eta_{\text{Hi}}+\gamma_{\text{Hik}}$$

where H_i_ is the value of the structural parameter H (V, CL, Q, ka) for a given individual i, H_pop_ is the value estimated for the population, BW is the bodyweight of an individual i, β_BW_ is the value for the relationship with the bodyweight normalized to the median value of all pigs (31.1 kg), and η_Hi_ and ɣ_Hik_ are the random effects for the IIV and IOV, respectively.

Potential correlations between the random effects were also estimated. These correlations are important for the simulating a large cohort of individuals to avoid unrealistic variability that may be greater than the reality.

Plasma concentrations were log transformed and the residual error was best described by a constant model for all drugs.

An internal validation was performed based on the predicted-corrected visual predictive check (pcVPCs) with 500 simulations (Bergstrand et al. 2011) and stratified by route of administration.

### External validation and simulations of the TMP/S ratios

The final popPK models, including all the estimated variabilities (IIV and IOV), the relevant covariate relationships, the correlations between random effects and their associated uncertainties (obtained after bootstrap analysis), were exported from Monolix^®^ to Simulx^®^ to generate Monte Carlo simulations for different purposes.

First, the predictive ability of our popPK models was assessed against independent datasets (i.e. PK data that were not used for model development), as an external validation (Cheng et al. 2021). All the data used for the different external validations were mean plasma concentrations extracted using WebPlotDigitizer (version 5) from the studies of Baert et al. (Baert et al. 2001), Mengelers et al. (Mengelers et al. 1995) and Nouws et al. (Nouws et al. 1991). Simulations (*n* = 50 000) were done based on the experimental design of their study (i.e. doses, animal weight and administration routes) and extracted data were plotted against the 5-95% prediction intervals of these simulations. For TMP/SDZ, the experimental design of Baert et al. (Baert et al. 2001) was an oral administration of 25 mg/kg of SDZ and 5 mg/kg of TMP to pigs with a mean weight of 24 kg +/-4 kg. For TMP/SDMX, the experimental design of Mengelers et al. (Mengelers et al. 1995) was an IV administration of 25 mg/kg of SDMX and 5 mg/kg of TMP to pigs with a mean weight of 35.5 kg +/- 1.5 kg. For TMP/SMX, the experimental designs were an IV administration of 25 mg/kg of SDX and 5 mg/kg of TMP to pigs with a mean weight of of 37.5 +/- 2.5 kg (Mengelers et al. 1995) and an IV administration of TMP/SMX of 40 mg/kg of SMX and 8 mg/kg of TMP to pigs with a mean weight of 27.5 kg +/- 1.5 kg (Nouws et al. 1991).

Secondly, the impact of the protein binding was assessed by plotting the evolution of the predicted individual TMP/S ratios over 24h based on the total and effective (i.e. unbound) TMP/S concentrations for each pig from this study used to develop the popPK models. For this purpose, linearity of the binding was assumed over the range of concentrations studied, and the total plasma concentrations were corrected with the mean value of protein binding determined in this study (without variability as recommended by Toutain et al. (Toutain et al. 2023).

Finally, to generalize our results to pig production, Monte Carlo simulations were generated for a population of pigs (*n* = 50,000) with the same bodyweight range as the animals in our study and using the maximum recommended dosing regimens for oral use as described in the summary of product characteristic (SCP) for each combination (25 mg/kg of SDZ or SMX + 5 mg/kg of TMP and 37.36 mg/kg of SDMX + 8 mg/kg of TMP). The simulated (free) concentrations were then used to compute the (free) TMP:S ratio over 24 h and over the maximal duration of treatment (i.e. 5 or 7 days) for comparison with the targeted 1:19 ratio. Additionally, a less stringent ratio interval of 1:10 to 1:50 was used to interpret the results (see discussion).

## Results

### PK data after administration of TMP/SDZ, TMP/SMX and TMP/SDMX

**Figure 1.**
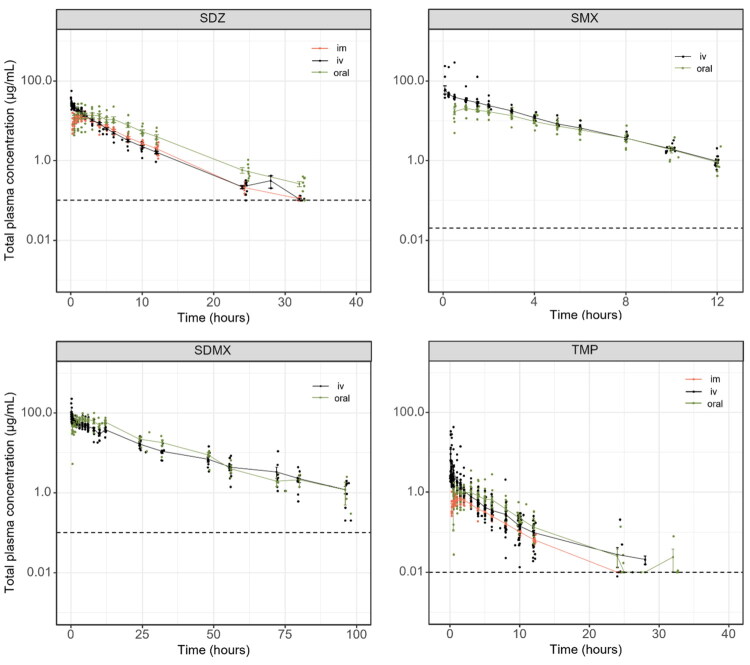
Scatter plot of the total plasma concentration of SDZ, SMX, SDMX and TMP versus time following a single IV (black), oral (green) or IM (red) administration. All individuals included in the modeling from our study (De smet’s data (De Smet et al. 2017) are not shown) are represented and each dot corresponds to one sampling. The mean total antibiotic concentration following the IV (black), oral (green) or IM (red) is represented by a solid line and error bars represent the standard error of the mean. The limit of quantification (LOQ) for SDMX and SDZ is 0.1 µg/mL, the LOQ for TMP is 0.01 µg/mL and the LOQ for SMX is 0.02 µg/mL. LOQs are represented in with black dashed line.

Animals showed no signs of distress following the IV, oral or IM administration for any of the three TMP/S combinations. Raw individual plasma concentrations of SDZ, SMX, SDMX and TMP versus time after dosing and the mean total concentrations are shown in Figure 1. Concentrations of SDZ, SDMX and TMP were detected above the limit of quantification up to 36, 96 and 30 h respectively. The assay results for SMX showed unexpected and unrealistic concentration values from 12h until the last sampling (no decrease in concentrations). Therefore, and based on previously reported T_1/2_ (between 2.3 and 3.1 h) (Nouws et al. 1991; Mengelers et al. 1995) SMX concentrations after 12h were discarded and not included in the modeling. Individuals (*n* = 1 for TMP/SDMX and *n* = 2 for TMP/SMX) or data (*n* = 155 outliers out of 1858 data included in the modeling) for which unexpected events occurred during sampling (mainly catheter issues) or obvious inconsistencies in drug concentrations were considered as outliers.

#### Population PK analysis

**Figure 2.**
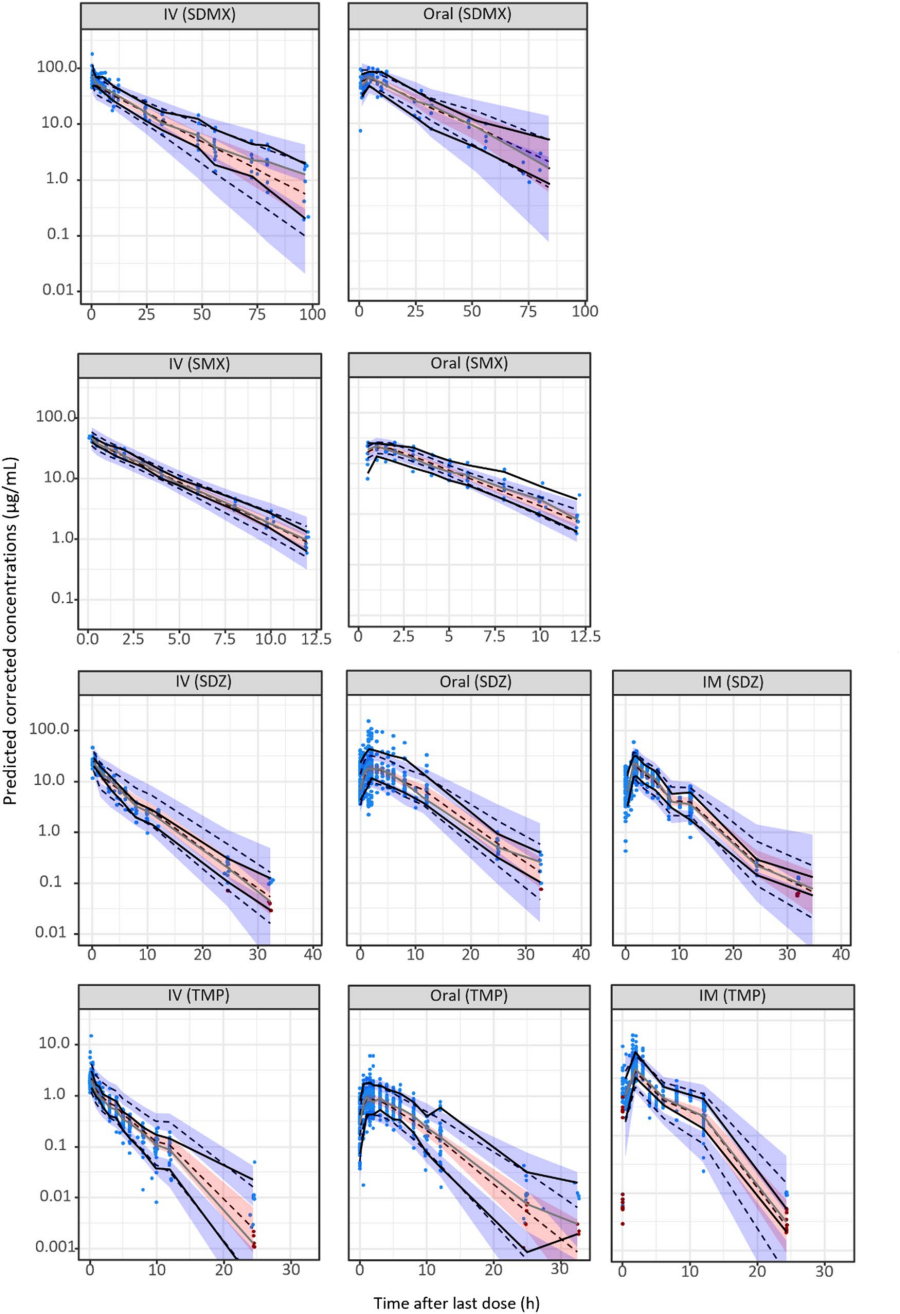
Predicted-corrected VPCs SDMX, SMX, SDZ and TMP following an IV (first column), oral (second column) or IM (third column) administration. Experimental data from this study and the study of De Smet are represented in blue dots, data below the LOQ in red, the empirical percentile is represented in a solid black line and the predicted percentile is represented in black dashed line. The 10% and 90% intervals are represented by the upper and lower blue areas and the median is represented by the pink area.

According to the BICc comparison and the goodness of fit plots (PWRES, IWRES and NPDE, Figure S1), a 2-compartment structural model was found to best fit the concentrations over time for SMX, SDMX, SDZ and TMP. The values of the median of the population parameters (V1, V2, CL, Q, F and ka), the standard deviations of the random effects and the error model for each molecule are presented in Table 2. The IIV and IOV for Q (and ka only for SMX) were fixed to 0 as our data did not allow an accurate prediction of the variability associated with these typical parameters. Details on the values of the correlations between the random effects can be found in the (Supplementary Data Table S6). The estimated clearances were different according to the sulfonamide (0.015 L/h/kg for SDMX, 0.12 L/h/kg for SDZ and 0.21 L/h/kg for SMX) but remained lower than the clearance of TMP (0.48 L/h/kg). Bodyweight was found to have a positive influence on the clearance of TMP and SDZ and explained part of their IOV. The volume of the central compartment (V1) of SDMX was found to be the lowest among the three sulfonamides studied with a value of 0.13 L/kg compared to 0.3 L/kg for SDZ and 0.48 L/kg for SMX. Trimethoprim had a V1 about two times higher (0.92 L/kg) than SMX, and again, bodyweight had a positive influence on the V1 of TMP and SDZ and explained part of their IOV. For the peripheral volume (V2), SDMX and SMX shared a similar value (0.16 L/kg for SDMX and 0.17 L/kg for SMX), which is about 5 times lower compared to the value for TMP (0.86 L/kg). The elimination half-life and the AUC_∞_ were calculated for each molecule and route of administration and are shown in Table 3. Overall, all the models developed gave a good fitting of the data as shown by the pcVPCs (Figure 2) which showed only minor misspecification. For the external validation of the popPK models for SDMX, SDZ and TMP, extracted data from the literature (Nouws et al. 1991; Mengelers et al. 1995; Baert et al. 2001) were actually included in the 5-95% prediction interval strengthening the good predictivity of these models (Figure S2). For SMX, our model underpredicted the 5-95% interval as the extracted data from Mengelers et al. (Mengelers et al. 1995) and Nouws et al. (Nouws et al. 1991) were above the simulated 95% percentile.

**Table 2.** Values of the median of the population parameters, the standard deviations of the random effects and the residual error variability for each model using nonlinear mixed effect (NLME) following a single IV, oral and IM administration of TMP/SDZ, TMP/SDMX or TMP/SDZ to pigs.

Fixed effects		
Parameters	Units	Median [2.5%-97.5%]
F_SDZ_oral	%	93 [0.82–0.98]
F_SDMX_oral	%	68 [0.59–0.79]
F_SMX_oral	%	64 [0.54–0.78]
F_TMP_oral F_SDZ_IM F_TMP_IM	% % %	58 [0.51–0.67] 99 [0.99 − 0.99] 99 [0.99 − 0.99]
ka_SDZ	h^-1^	0.50 [0.39–0.81]
ka_SDMX	h^-1^	0.60 [0.42–0.86]
ka_SMX	h^-1^	1.82 [1.22–2.86]
ka_TMP	h^-1^	0.66 [0.42–0.85]
ka_SDZ_IM	h^-1^	1.3 [0.96–1.65]
ka_TMP_IM	h^-1^	1.14 [0.87–1.45]
CL_SDZ	L/h/kg	0.12 [0.11–0.13]
CL_SDMX	L/h/kg	0.015 [0.014–0.017]
CL_SMX	L/h/kg	0.21 [0.19–0.23]
CL_TMP	L/h/kg	0.48 [0.45–0.52]
V1_SDZ	L/kg	0.3 [0.26–0.36]
V1_SDMX	L/kg	0.13 [0.091–0.19]
V1_SMX	L/kg	0.48 [0.41–0.53]
V1_TMP	L/kg	0.92 [0.78–1.08]
Q_SDZ	L/h/kg	0.32 [0.16–0.87]
Q_SDMX	L/h/kg	0.20 [0.049–0.37]
Q_SMX	L/h/kg	0.83 [0.42–1.63]
Q_TMP	L/h/kg	1.26 [0.64–2.10]
V2_SDZ	L/kg	0.29 [0.22–0.35]
V2_SDMX	L/kg	0.16 [0.12–0.20]
V2_SMX	L/kg	0.17 [0.10–0.23]
V2_TMP	L/kg	0.86 [0.72–1.04]
β_CL_SDZ_BW	–	0.51 [0.38–0.64]
β_CL_TMP_BW	–	0.78 [0.58–0.98]
β_V1_SDZ_BW	–	1.1 [0.71–1.38]
β_V1_TMP_BW	–	1.33 [0.90–1.84]
Standard deviations of the Random Effects		
		Median
𝜔_ka_SDZ		0.52 [0.34–0.72]
𝜔_ka_SDMX		0.37 [0.21–1.03]
𝜔_ka_TMP		0.63 [0.39–0.85]
ɣ_CL_SDZ		0.33 [0.23–0.47]
ɣ_CL_SDMX		0.18 [0.13–0.25]
ɣ_CL_TMP		0.34 [0.30–0.40]
ɣ_V1_SDZ		0.12 [0.06–0.22]
ɣ_V1_SDMX		0.40 [0.19–0.63]
ɣ_V1_SMX		0.09 [0.05–0.15]
ɣ_V1_TMP		0.44 [0.30–0.57]
ɣ_V2_SDZ		0.62 [0.45–0.85]
ɣ_V2_SDMX		0.34 [0.18–0.56]
ɣ_V2_SMX		0.40 [0.22–0.67]
ɣ_V2_TMP		0.42 [0.30–0.56]
Error Model Parameters		
a_SDZ		0.32 [0.25–0.39]
a_SDMX		0.22 [0.21–0.24]
a_SMX		0.18 [0.13–0.22]
a_TMP		0.40 [0.33–0.46]

Abbreviations: F = bioavailability; ka = absorption rate constant; V1 = volume of the central compartment; V2 = volume of the peripheral compartment; Cl = clearance; β_V_SDZ_BW = covariate effect of “BW”; TMP = trimethoprim; SDZ = sulfadiazine; SDMX = sulfadimethoxine; SMX = sulfamethoxazole; P2.5 and P97.5 = confidence intervals; 𝜔 = value of the inter-individual variability (IIV); ɣ = value of the inter-occasion variability (IOV); a = constant residual error.

**Table 3.** Mean values of the terminal elimination half-life and the area under the curve for SDZ, SDMX, SMX, TMP using nonlinear mixed effect (NLME) following a single administration of TMP/SDZ, TMP/SDMX or TMP/SMX in pigs.

	Route of administration			
	IV		Oral (gavage)	
Parameters	Mean values	Range (min-max)	Mean values	Range (min-max)
T_1/2β_ SDZ (h)	3.7	3.1 − 4.2	4.4	3.2 − 6.0
T_1/2β_ SDMX (h)	14.8	10.9 − 20.2	13.6	9.8 − 16.5
T_1/2β_ SMX (h)	2.2	1.9 − 2.5	2.3	1.8 − 3.2
T_1/2β_ TMP (h)	2.9	1.2 − 3.7	3.5	1.8 − 5.8
AUC_∞_ SDZ (h x µg/mL)	99.2	74.6 − 113.8	144.3	87.2 − 220.6
AUC_∞_ SDMX (h x µg/mL)	1280.3	916.5 − 1709.4	1629.0	1048.7 − 2215.8
AUC_∞_ SMX (h x µg/mL)	140.93	140.92 − 140.93	92.39	92.39 − 92.39
AUC_∞_ TMP (h x µg/mL)	6.9	3.1 − 10.7	7.3	3.3 − 18.2

Abbreviations: T_1/2β_ = terminal elimination half-life; AUC_∞_ = area under the curve from time 0 to infinity; IV = intravenous; TMP = trimethoprim; SDZ = sulfadiazine; SDMX = sulfadimethoxine; SMX = sulfamethoxazole. Means are mean values of all individuals.

#### Protein binding experiment

The average *in vivo* protein binding calculated in this study was 57.3% (± 7.9) for SMX, 94.1% (± 3.1) for SDMX, 29.2% (± 9.1) for SDZ and 51.2% (± 9.7) for TMP. The total concentrations were then corrected by the corresponding unbound fraction (fu) (i.e. 42.7% for SMX, 5.9% for SDMX, 70.8% for SDZ and 48.9% for TMP) to obtain the free concentration ratios of TMP/S over time used for simulation purposes.

#### Simulations

**Figure 3.**
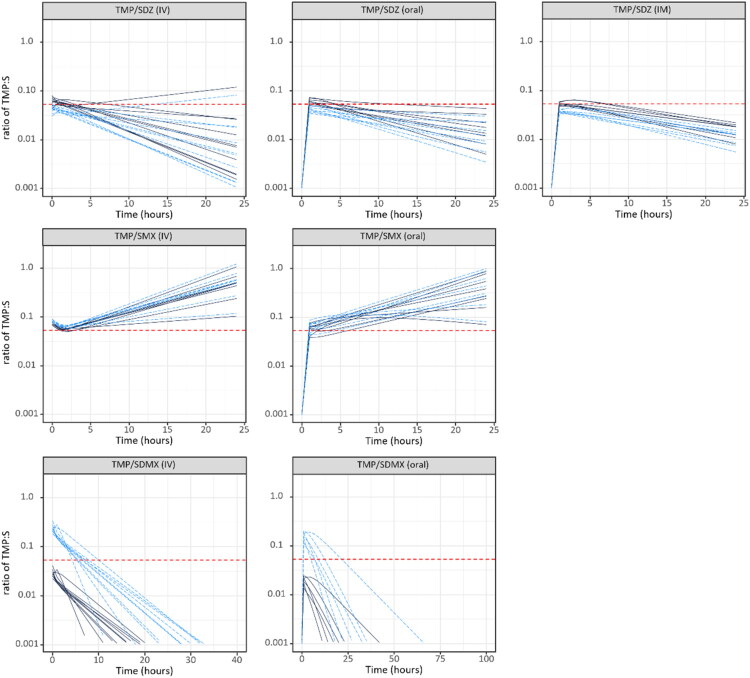
Scatter plot of the predicted individual total and unbound ratios of TMP/SDZ, TMP/SMX and TMP/SDMX following a single IV, oral or IM (only TMP/SDZ) administration at the doses used in the study over 24 h or 96 h (only SDMX). All individuals included in the modeling from our study (De smet’s data are not shown) are represented, the total concentration ratio is represented with black lines and the unbound concentration ratio is represented with dotted blue lines. The 1:19 ratio is represented by the red dotted line.

**Figure 4.**
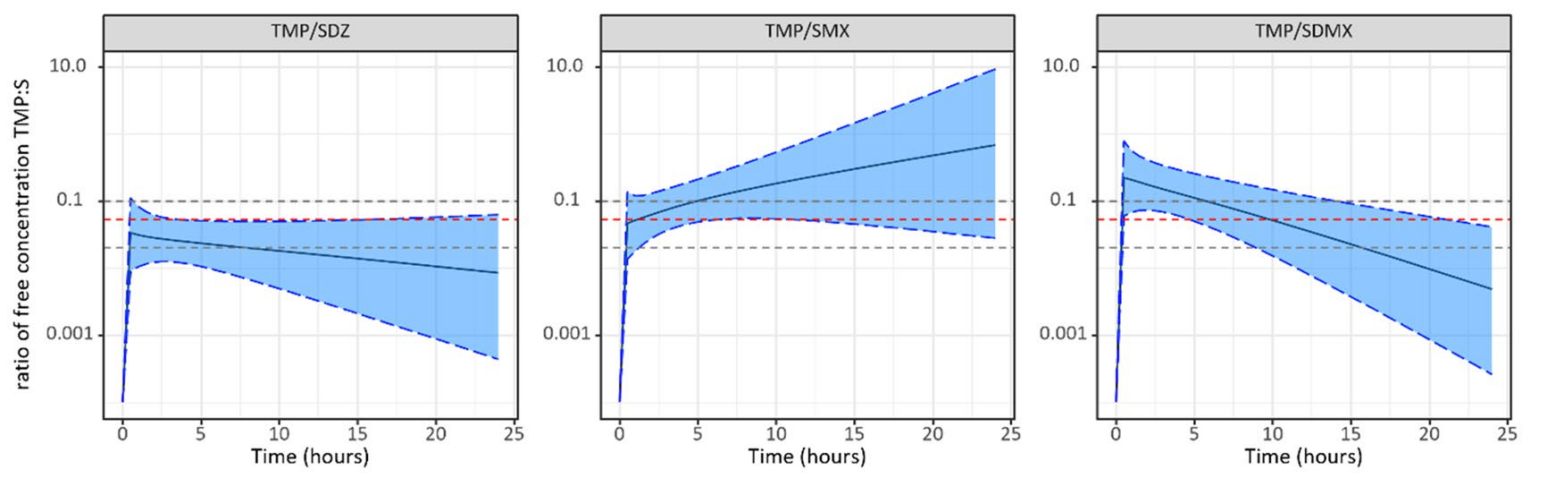
Simulation of the ratio of free concentrations of TMP/SDZ, TMP/SMX and TMP/SDMX over 24 h following a single oral administration (*n* = 50 000 pigs). The median is represented by a solid black line and the blue shaded area represents the 5^th^ and 95^th^ percentiles. The 1:19 ratio is represented by a dotted red line.

Based on the Empirical Bayes Estimates (EBEs) from the final popPK models and the determined average fu, the individual total and unbound concentration ratio profiles were computed for each pig from this study over 24h after IV, oral and IM administrations (Figure 3). The individual ratios showed that the TMP/SDZ combination seems to be closer to the targeted 1:19 plasma concentration than the other two combinations. The same approach was then widened to a much larger population (*n* = 50,000): the ratio of the unbound concentrations of TMP/SDZ, TMP/SDMX and TMP/SMX over 24h after an oral administration at the registered doses was simulated based on the estimated PK parameters (typical values, variabilities and correlations) (Figure 4). The unbound concentration ratio of TMP/SDMX and TMP/SMX varies greatly over time, but for the combination TMP/SDZ it remains fairly constant over 24h. Moreover, the targeted 1:19 ratio for TMP/SDMX and TMP/SMX is rarely achieved (or in a very small proportion of pigs), as the computed ratio consistently deviates and is either lower or higher, respectively. When applying a broader ratio range of 1:10 to 1:50, the proportion of simulated pigs remaining within this interval over 24h was: 8.8% for the TMP/SMX combination, 46.8% for the TMP/SDZ combination, and 76.5% for the TMP/SDMX combination. The median duration of the TMP:S ratio within the same interval was 4.5h (i.e. 18.8%) for the TMP/SMX combination, 14h (i.e. 58.3%) for the TMP/SDZ combination and 12h (50%) for the TMP/SDMX combination over 24h.

## Discussion

The aim of this study was to obtain individual pharmacokinetic data following a single IV, oral, or IM administration of sulfonamide drugs (SDZ, SDMX and SMX) commonly administered in combination with TMP in pigs and to determine the *in vivo* protein binding of each molecule. In addition, the goal was to compare the ratio of unbound concentrations obtained in pigs to the targeted concentration ratio of 1:19, the standard used for antimicrobial susceptibility testing of TMP/S.

First, popPK modeling was used to fit the PK data and a 2-compartmental model gave a better fit for SMX, SDMX, SDZ and TMP. All drugs could be considered as bioavailable following an oral administration with F values of 58% for TMP, 64% for SMX, 68% for SDMX and 93% for SDZ. The bioavailability of SDZ and TMP was close to 100% following an IM administration. The inter-individual variability of the absorption rate constant (ka) was high and varies between molecules. However, with the authors experiences, we found this parameter usually difficult to estimate.

The results of the external validation highlighted the good predictive ability of the developed population PK models for SDMX, SDZ and TMP, increasing the confidence in the popPK models developed in this study to predict drug behavior at the population level in pigs. However, the popPK model for SMX slightly underpredicted the extracted data from the literature. The difference in estimated clearance values between our study and those reported in the studies by Mengelers et al. (Mengelers et al. 1995) and Nouws et al. (Nouws et al. 1991) could be an explanation. Clearance is a crucial PK parameter as it governs the overall drug exposure in an individual (Toutain and Bousquet-Mélou 2004b). In our study, the clearance of SMX, determined by NLME was higher (0.21 L/h/kg) compared to the mean clearances reported in the other two studies (0.13 L/h/kg and 0.1 L/h/kg) (Nouws et al. 1991; Mengelers et al. 1995). A possible explanation could be a sex-effect, as Mengelers et al. (Mengelers et al. 1995) used castrated males compared to females in our study (no information on the sex of the animals was reported in Nouws et al. (Nouws et al. 1991)). Moreover, extracting the mean data from Mengelers et al. (Mengelers et al. 1995) was challenging due to the poor quality of the graphs, which may have introduced a slight bias when plotting against the 95% prediction interval (Li et al. 2015). However, the T_1/2β_ values were similar as outlined by the parallel terminal slopes (Figure S2). Sulfadimethoxine has the lowest clearance (0.015 L/h/kg) compared to the other two sulfonamides (0.12 L/h/kg for SDZ and 0.21 L/h/kg for SMX) and also the longest T_1/2β_ (14.8 h). This T_1/2β_ value is in good agreement with the value (12.5 +/- 1.3 h) obtained in a recent PK study in pigs receiving TMP/SDMX *via* medicated feed (Santos-Santórum Suárez et al. 2025). Trimethoprim has the highest clearance value (0.48 L/h/kg) which is also in accordance with previous publications (Nielsen and Gyrd-Hansen 1994; Baert et al. 2001). It is interesting to note that the inter-occasion variability in clearance was low to moderate (0.18–0.34), suggesting a relatively similar elimination within the same individual across different occasions. It is also attributed to the inclusion of the bodyweight as a covariate. The bodyweight was found to have a positive influence on the clearance and V1 of SDZ and TMP, meaning that the typical values for these two drugs would be higher in heavier pigs. For instance, the CL values vary from 0.12 L/h/kg for SDZ and 0.48 L/h/kg for TMP (for a typical individual pig of 31.1 kg in our study) to 0.14 L/h/kg and 0.60 L/h/kg, respectively, for a pig of 41.1 kg. The V1 values vary from 0.30 L/kg for SDZ and 0.92 L/kg for TMP again for a typical pig of 31.1 kg to 0.41 L/kg and 1.33 L/kg respectively for a pig of 41.1 kg. This highlights the strength of popPK modeling to identify relevant biological covariates even when the data come from different studies with unbalanced designs (Bon et al. 2018). To this end, meta-analysis of existing raw data is crucial in veterinary medicine and especially for the optimization of antimicrobial dosing regimens (Li et al. 2015; Toutain et al. 2017).

The relevance of determining the plasma protein binding is that only the free fraction (or unbound fraction) of the drug can be efficient on pathogens (Wanat 2020). The average protein binding determined for each drug in this study varies widely according to the molecule (from 29.24% for SDZ to 94.07% for SDMX) and is in consistent with values reported in the literature (Nouws et al. 1989, 1991; Mengelers et al. 1995). First, predictions of the individual total and unbound concentration ratios over time following IV, oral or IM administrations in pigs included in our study showed that this ratio varies greatly between TMP/S combinations, but also between individuals who received the same TMP/S combination (Figure 3). The second major step in this study was to perform simulations with the final popPK models to estimate the distribution of the unbound TMP:S ratios in pigs over time following an oral administration at the population (or herd) level. As a result, simulations at the population level (*n* = 50,000) showed very different profiles depending on the TMP/S combination. The main finding of this study is that the target ratio of 1:19 is rarely achieved, and when it is, it is transient and occurs in a very small fraction of the pigs. Moreover, depending on the drug, the ratio can be higher, perhaps with a greater influence of TMP while for others it was lower, with a lesser influence of TMP on the bacteria. We also chose to compare with a wider ratio interval of 1:10-1:50 (see below). Even with this extended interval, simulations for the TMP/SMX combination indicated that only a small proportion of individuals (8.8% over 24 h) would maintain an *in vivo* ratio within this range. This is also reflected in the low median duration of the ratio remaining within the interval (4.5 h over 24 h). This is consistent with the T_1/2β_ values determined: since SMX has a shorter T_1/2β_ compared to TMP, the ratio increases over time. The unbound concentration ratio of TMP/SDZ is more constant over time in pigs due to the similar T_1/2β_ values for both drugs (3.7 h for SDZ and 2.9 h for TMP). This combination, out of the three tested, stays closest to the 1:19 ratio over time: the median duration of the TMP:S ratio within the 1:10 − 1:50 interval is 14 h (58.33% over 24 h), with approximately 46.8% of the simulated pigs maintaining this ratio. Finally, for the combination TMP/SDMX, the median duration between the 1:10 and 1:50 ratios is similar (12 h, i.e. 50%) to the median duration for TMP/SDZ and in a larger proportion of simulated pigs (76.5% compared to 46.8% for TMP/SDZ). However, due to the large differences in the T_1/2β_ values (14.8h for SDMX and 2.9h for TMP), daily administrations will lead to an increase in SDMX concentrations over time (plasma accumulation). Consequently, the concentration ratio will decrease after each new administration (as shown in Figure S3).

Overall, these simulations suggest that if the aim of current TMP/S dosing regimens is to achieve an *in vivo* ratio of 1:19 in pigs, they are inadequate, regardless of the combination chosen. This is similar to our recent study with the same TMP/S combinations in poultry (Boulanger et al. 2024). However, even if the targeted 1:19 ratio is not observed, this would not imply a sudden loss of synergism of the combination in the presence of other ratios. In fact, the “optimal” synergistic ratio seems to depend on the strain and the sulfonamides used in the combination, and is therefore very variable (Bushby 1980). Indeed, preliminary data from our team showed that for *Escherichia coli*, the optimal TMP:S ratio could vary depending on both to the bacterial strain and to the sulfonamide used in the combination, as synergistic ratios varied from 1:32 to 1:320 for SMX, from 1:16 to 1:1,280 for SDZ, and from 1:64 to 1:4,096 for SDMX (Boulanger et al. 2023). Due to the large variability among TMP/S ratios in pigs pointed out by our simulations, it would be interesting to better understand the pharmacodynamic (PD) effects of these antibiotics in combination (for this large range of observed TMP/S ratios) against common pathogens in pigs. This is a challenge that our team is currently investigating by developing a semi-mechanistic PK/PD model based on time-kill curve experiments for various bacteria species (Wicha et al. 2017). This PK/PD approach would help to determine whether the 1:5 dose ratio actually administered could be effective or whether there is a need to adjust and optimize the dosing regimens of TMP/S in pigs. Furthermore, achieving the optimal drug exposure *in vivo* is critical for both animals and humans, as suboptimal concentrations of antibiotics have been shown to trigger the selection of resistance in pathogenic and commensal bacteria that can spread into the environment (Lees et al. 2019; Dréano et al. 2022).

An important limitation of this study is the use of gavage for the oral administration, which is not representative of the current farming practice, but was the most convenient to control the exact doses administered. Pigs are usually treated *via* drinking water or medicated feed, but individual water/feed intake is often unmonitored and variable, which could result in greatly increased inter-individual variability regarding exposure (Hernández et al. 2024).

In our study, the 1:19 ratio cited as a clinical target in human medicine following administration of a 1:5 TMP/S dose was not achieved, and similar findings have been reported in many other animal species (Mengelers et al. 1995; Baert et al. 2001; Ferran et al. 2020; Boulanger et al. 2024). This suggests that clinical targets derived from human medicine may not always be directly transferable or applicable to veterinary contexts. Differences in species-specific pharmacokinetics, drug–host and drug–pathogen interactions, as well as unique physiological characteristics of each animal, can influence both the attainment and the clinical relevance of such targets (Lees and Shojaee Aliabadi 2002). In conclusion, this study shows that the *in vivo* TMP/S concentration in pigs varies widely depending on the sulfonamides used in the combination and is rarely equal to 1:19, questioning the relevance of the 1:5 TMP:S dose ratio used in almost all veterinary formulations. Combining the current PK model with future PD data in a PK/PD modeling approach would be of great help to preserve a safe and optimal efficacy of this important antimicrobial combination in veterinary medicine.

## Supplementary Material

Supplementary_materials.docx

## Data Availability

All PK data collected in this study for the manuscript has been published and is available (except data from De Smet’s study) here: https://doi.org/10.57745/Q2H4LQ. Part of this work has been presented at the 15th International Congress of the European Association for Veterinary Pharmacology and Toxicology held Bruges, Belgium, July 2–5, 2023.
