# Supplementary material for: Population pharmacokinetic modeling of sulfadimethoxine, sulfadiazine and sulfamethoxazole combined to trimethoprim in pigs
Source: Vet Q. 2025 Sep 29;45(1):2565351. doi: 10.1080/01652176.2025.2565351 (PMC12481524; doi:10.1080/01652176.2025.2565351)
Supplement: Supplementary_materials.docx [file TVEQ_A_2565351_SM2231.docx]

**Supplementary materials**

Supplementary data Figure 1: Graphical evaluation with of the SDMX (A), SDZ (B), SMX (C) and TMP (D) final models. Plot of the population weighted residuals (PWRES) is on the left, of the individual weighted residuals (IWRES) is in the middle and of the normalized prediction distribution errors (NPDE) is Data are represented with blue dots, censured data are represented with red dots and the spline interpolation is represented with a yellow line.


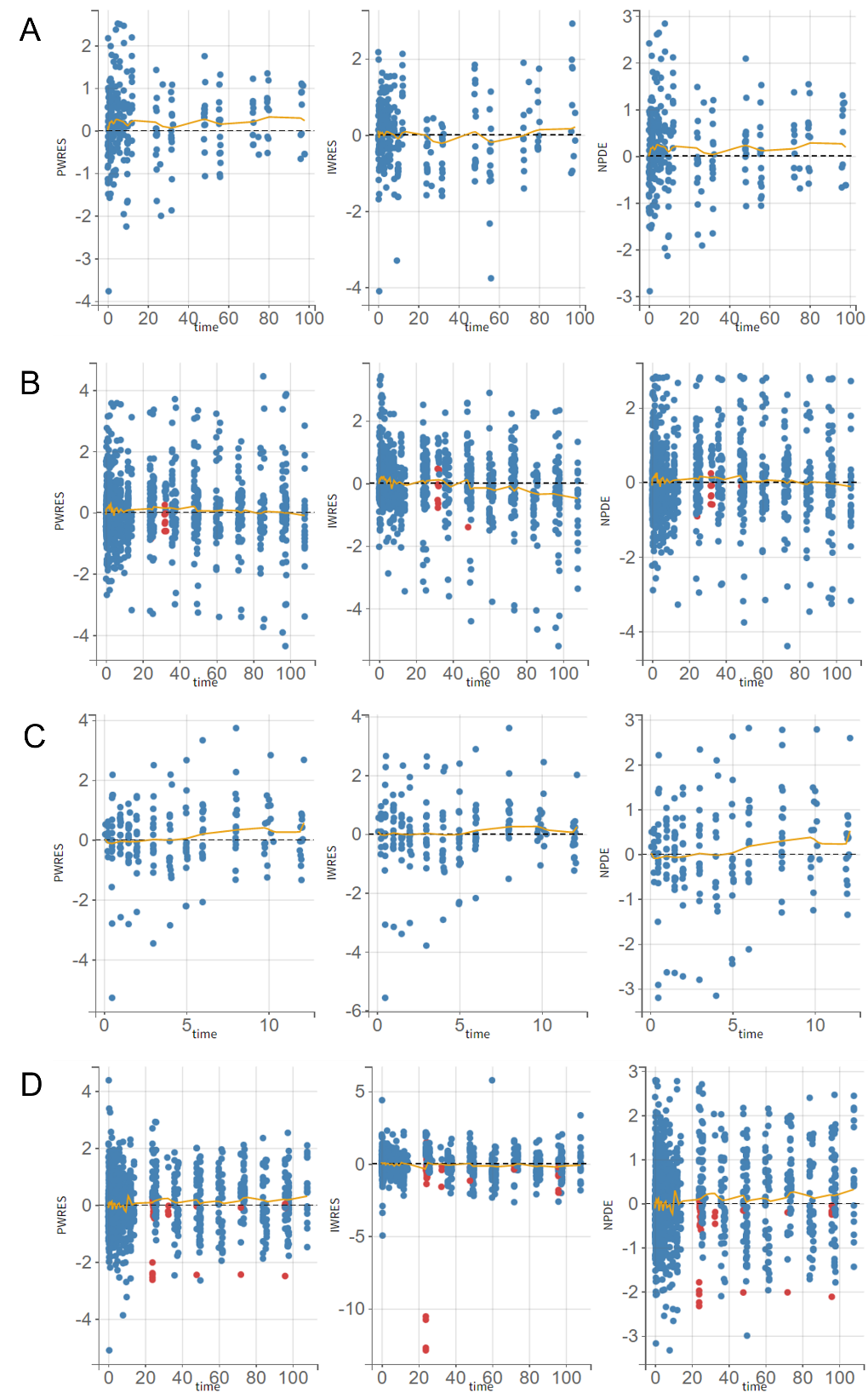


Supplementary data Figure 2: External validation of the final models using Baert et al. (A = SDZ, B = TMP), Mengelers et al. (C = SDMX, E = SMX, D and F = TMP) and Nouws et al. (G = SMX, H = TMP) studies. The median (n = 500 pigs) is represented with a solid black line and the 90% prediction interval with a blue area. Mean data from the respective studies are plotted as red dots.


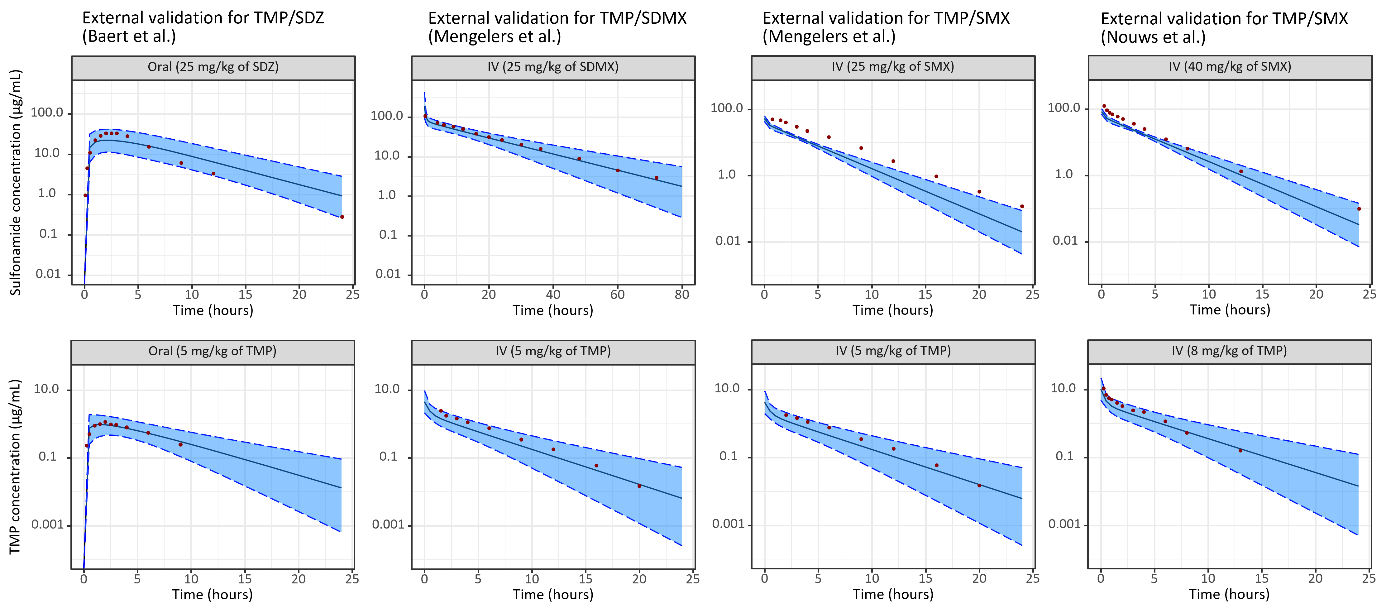


Supplementary data Figure 3: Simulation of the ratio of free concentrations of TMP/SDZ, TMP/SMX and TMP/SDMX following the dosing regimens (doses and time interval between doses) recommended on the SCP (25 mg/kg of SDZ or SMX + 5 mg/kg of TMP per day over 7 days and 37.36 mg/kg of SDMX + 8 mg/kg of TMP per day over 5 days). The median is represented by a solid black line and the blue shaded area represents the 5^th^ and 95^th^ percentiles. The 1:19 ratio is represented by a dotted red line.


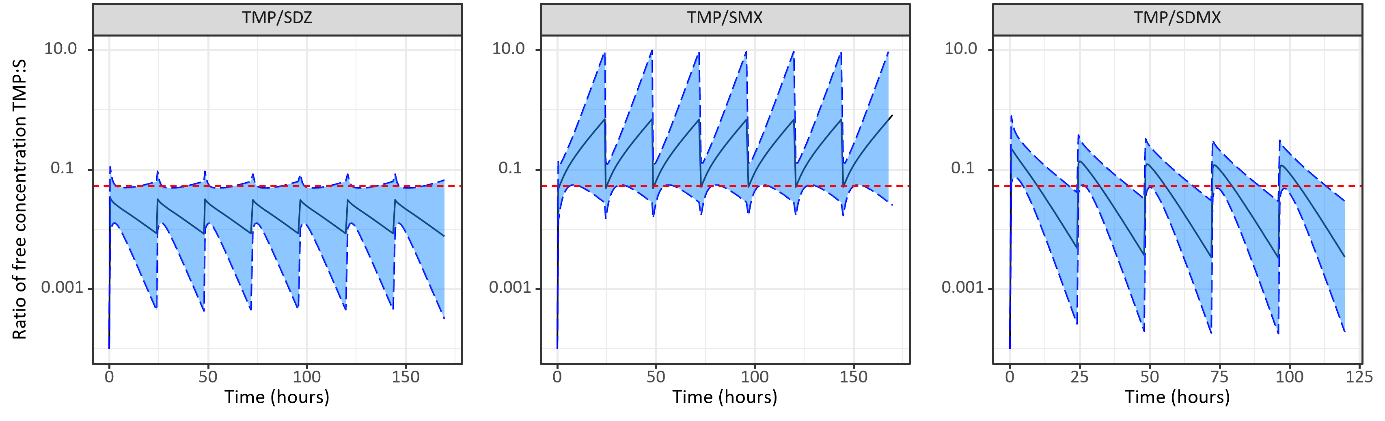


**Supplementary Tables**

Table S1. MRM transitions and MS/MS parameters for sulfadiazine (SDZ), sulfamethoxazole (SMX), sulfadimethoxine (SDMX), trimethoprim (TMP) and their respective labelled internal standards.

| **Analyte** | **Precursor**  **ion**  **(*m/z*)^a^** | **Product**  **ions**  **(*m/z*)** | **CE^b^**  **(eV)** | **Cone**  **(V)** | **Retention**  **time**  **(min)** |
| --- | --- | --- | --- | --- | --- |
| SDZ | 251.2 | 156.1^c^  92.1 | 15  25 | 30  30 | 1.53 |
| SDZ-d4 | 254.9 | 160.0^c^  96.0 | 15  25 | 30  30 | 1.50 |
| SMX | 254.1 | 156.0 ^c^  92.2 | 15  25 | 30  30 | 3.43 |
| SMX-d4 | 258.0 | 160.2 ^c^  96.2 | 15  25 | 40  40 | 3.41 |
| SDMX | 311.3 | 156.1^c^  92.1 | 20  30 | 30  30 | 4.18 |
| SDMX-d6 | 317.3 | 162.2^c^  92.2 | 20  30 | 30  30 | 4.13 |
| TMP | 291.3 | 230.1^c^  123.1 | 25  25 | 40  40 | 1.89 |
| TMP-d9 | 300.1 | 123.1^c^  234.3 | 25  25 | 40  40 | 1.81 |

^a^ m/z = mass to charge ratio, ^b^ CE = collision energy, ^c^ ion used for quantification.

Table S2. Results of the evaluation of calibration range (correlation coefficient (r), goodness-of-fit coefficient (gof), limit of detection (LOD) and lower llimit of quantification (LLOQ) for sulfadiazine (SDZ), sulfamethoxazole (SMX), sulfadimethoxine (SDMX) and trimethoprim (TMP) in pig plasma. Experiments were performed on 1 day.

| **Analyte** | **Spiked Calibration Range**  **(ng/mL)** | **r^a^** | **Gof^b^**  **(%)** | **LOD^c^**  **(ng/mL)** | **LOQ**  **(ng/mL)** |
| --- | --- | --- | --- | --- | --- |
| SDZ | 20 to 100000 | 0.9990 | 4.5 | 0.59 | 20 |
| SMX | 20 to 100000 | 0.9996 | 2.7 | 0.45 | 20 |
| SDMX | 20 to 100000 | 0.9994 | 3.4 | 0.61 | 20 |
| TMP | 4 to 4000 | 0.9983 | 3.4 | 0.31 | 4 |

Note: ^a^r ≥ 0.99; ^b^Gof ≤ 10 %; ^c^LOD : calculated concentration that corresponds to a signal-to-noise ratio (S/N) = 3

Table S3. Results of the evaluation of calibration range (correlation coefficient (r), goodness-of-fit coefficient (gof), limit of detection (LOD) and lower limit of quantification (LLOQ) for sulfadiazine (SDZ), sulfamethoxazole (SMX), sulfadimethoxine (SDMX) and trimethoprim (TMP) in filtered pig plasma that was used for protein binding determination. Experiments were performed over 3 days.

| **Analyte** | **Spiked Calibration Range**  **(ng/mL)** | **r^a^** | **Gof^b^**  **(%)** | **LOD^c^**  **(ng/mL)** | **LOQ**  **(ng/mL)** |
| --- | --- | --- | --- | --- | --- |
| SDZ | 20 to 100000 | 0.9994 ± 0.0002 | 3.1 ± 0.5 | 3.31 | 20 |
| SMX | 20 to 100000 | 0.9995 ± 0.0003 | 2.9 ± 0.8 | 1.33 | 20 |
| SDMX | 20 to 100000 | 0.9995 ± 0.0002 | 2.9 ± 0.5 | 1.68 | 20 |
| TMP | 4 to 4000 | 0.9982 ± 0.0016 | 5.0 ± 2.5 | 0.32 | 4 |

Note: ^a^r ≥ 0.99; ^b^Gof ≤ 10 %; ^c^LOD : calculated concentration that corresponds to a signal-to-noise ratio (S/N) = 3

Table S4. Results of the within-run precision and accuracy evaluation for the analysis of sulfadiazine (SDZ), sulfamethoxazole (SMX), sulfadimethoxine (SDMX) and trimethoprim (TMP) in pig plasma.

| **Analyte** | **Spiked concentration**  **(ng/mL)** | **Mean concentration ± SD**  **(ng/mL)** | **Precision, RSD**  **(%)** | **Accuracy**  **(%)** |
| --- | --- | --- | --- | --- |
| SDZ | 20.0 (n = 6) ^a^ | 17.7 ± 2.1 | 11.9 | -11.4 |
|  | 500.0 (n = 6) | 490.7 ± 32.0 | 6.5 | -1.9 |
|  | 5000.0 (n = 6) | 5042.8 ± 299.6 | 5.9 | 0.9 |
|  | 50000.0 (n = 6) | 51932.2 ± 2009.1 | 3.9 | 3.9 |
| SMX | 20.0 (n = 6) | 17.0 ± 1.2 | 6.9 | -15.0 |
|  | 500.0 (n = 6) | 506.9 ± 36.2 | 7.1 | 1.4 |
|  | 5000.0 (n = 6) | 4845.8 ± 94.7 | 2.0 | -3.1 |
|  | 50000.0 (n = 6) | 50133.5 ± 598.2 | 1.2 | 0.3 |
| SDMX | 20.0 (n = 6) | 19.3 ± 1.7 | 8.9 | -3.8 |
|  | 500.0 (n = 6) | 537.6 ± 26.7 | 5.0 | 7.5 |
|  | 5000.0 (n = 6) | 5008.8 ± 68.9 | 1.4 | 0.2 |
|  | 50000.0 (n = 6) | 46163.9 ± 785.8 | 1.7 | -7.7 |
| TMP | 4.0 (n = 6) | 3.8 ± 0.6 | 14.6 | -4.7 |
|  | 100.0 (n = 6) | 103.4 ± 3.0 | 2.9 | 3.3 |
|  | 1000.0 (n = 6) | 980.3 ± 23.5 | 2.4 | -2.0 |

Note: ^a^ Within-run accuracy and precision (n ≥ 5); ^b^ Between-run accuracy and precision (at least 3 analytical runs over at least 2 days); SD: standard deviation; RSD: relative standard deviation; Acceptance criteria for accuracy: LLOQ: ± 20 %, other concentration levels : ± 15 %; Acceptance criteria for precision (RSD_max_): LLOQ: ± 20 %, other concentration levels : ± 15 % [ICH M10].

Table S5. Results of the within-run and between-run precision and accuracy evaluation for the analysis of sulfadiazine (SDZ), sulfamethoxazole (SMX), sulfadimethoxine (SDMX) and trimethoprim (TMP) in filtered pig plasma that was used for protein binding determination. Experiments for the between-run precision and accuracy were performed over 3 days.

| **Analyte** | **Spiked concentration**  **(ng/mL)** | **Mean concentration ± SD**  **(ng/mL)** | **Precision, RSD**  **(%)** | **Accuracy**  **(%)** |
| --- | --- | --- | --- | --- |
| SDZ | 20.0 ^a^ (n = 6) | 20.0 ± 1.4 | 7.1 | -0.2 |
|  | 20.0 ^b^ (n = 18) | 18.6 ± 1.9 | 10.2 | -7.3 |
|  | 100.0 ^a^ (n = 5) | 89.2 ± 7.4 | 8.3 | -10.8 |
|  | 100.0 ^b^ (n = 17) | 92.6 ± 6.5 | 7.1 | -7.4 |
|  | 1000.0 ^a^ (n = 5) | 1004.4 ± 31.0 | 3.1 | 0.4 |
|  | 1000.0 ^b^ (n = 16) | 969.5 ± 50.0 | 5.2 | -3.1 |
|  | 10000.0 ^a^ (n = 6) | 10355.2 ± 212.3 | 2.1 | 3.6 |
|  | 10000.0 ^b^ (n = 18) | 9642.3 ± 614.6 | 6.4 | -3.6 |
|  | 50000.0 ^a^ (n = 6) | 52630.9 ± 1302.9 | 2.5 | 5.3 |
|  | 50000.0 ^b^ (n = 18) | 49289.4 ± 2798.0 | 5.7 | -1.4 |
| SMX | 20.0 ^a^ (n = 6) | 19.5 ± 0.6 | 3.0 | -2.4 |
|  | 20.0 ^b^ (n = 18) | 18.6 ± 1.8 | 9.6 | -7.0 |
|  | 100.0 ^a^ (n = 6) | 96.5 ± 9.6 | 9.9 | -3.5 |
|  | 100.0 ^b^ (n = 18) | 97.3 ± 7.0 | 7.2 | -2.7 |
|  | 1000.0 ^a^ (n = 6) | 1013.8 ± 26.5 | 2.6 | 1.4 |
|  | 1000.0 ^b^ (n = 16) | 1012.6 ± 33.0 | 3.3 | 1.3 |
|  | 10000.0 ^a^ (n = 6) | 9789.5 ± 151.7 | 1.5 | -2.1 |
|  | 10000.0 ^b^ (n = 18) | 9897.7 ± 263.0 | 2.7 | -1.0 |
|  | 50000.0 ^a^ (n = 6) | 49877.1 ± 2122.4 | 4.3 | -0.2 |
|  | 50000.0 ^b^ (n = 18) | 50391.2 ± 1483.6 | 2.9 | 0.8 |
| SDMX | 20.0 ^a^ (n = 6) | 19.6 ± 1.8 | 9.0 | -1.8 |
|  | 20.0 ^b^ (n = 18) | 19.2 ± 1.8 | 9.3 | -3.8 |
|  | 100.0 ^a^ (n = 6) | 102.9 ± 4.3 | 4.2 | 2.9 |
|  | 100.0 ^b^ (n = 17) | 98.7 ± 5.2 | 5.3 | -1.3 |
|  | 1000.0 ^a^ (n = 6) | 1023.9 ± 9.4 | 0.9 | 2.4 |
|  | 1000.0 ^b^ (n = 16) | 1008.5 ± 21.8 | 2.2 | 0.8 |
|  | 10000.0 ^a^ (n = 6) | 9889.1 ± 90.9 | 0.9 | -1.1 |
|  | 10000.0 ^b^ (n = 18) | 9733.2 ± 223.9 | 2.3 | -2.7 |
|  | 50000.0 ^a^ (n = 6) | 50555.5 ± 2176.2 | 4.3 | 1.1 |
|  | 50000.0 ^b^ (n = 18) | 49531.8 ± 1595.7 | 3.2 | -0.9 |
| TMP | 4.0 ^a^ (n = 6) | 4.2 ± 0.4 | 10.3 | 4.5 |
|  | 4.0 ^b^ (n = 18) | 4.0 ± 0.5 | 12.4 | -0.7 |
|  | 20.0 ^a^ (n = 6) | 19.5 ± 1.8 | 9.2 | -2.3 |
|  | 20.0 ^b^ (n = 18) | 20.0 ± 1.4 | 7.2 | 0.2 |
|  | 200.0 ^a^ (n = 6) | 200.6 ± 7.9 | 3.9 | 0.3 |
|  | 200.0 ^b^ (n = 16) | 204.6 ± 9.7 | 4.7 | 2.3 |
|  | 2000.0 ^a^ (n = 6) | 1889.0 ± 59.9 | 3.2 | -5.6 |
|  | 2000.0 ^b^ (n = 18) | 1915.2 ± 89.5 | 4.7 | -4.2 |

Note: ^a^ Within-run accuracy and precision (n ≥ 5); ^b^ Between-run accuracy and precision (at least 3 analytical runs over at least 2 days); SD: standard deviation; RSD: relative standard deviation; Acceptance criteria for accuracy: LLOQ: ± 20 %, other concentration levels : ± 15 %; Acceptance criteria for precision (RSD_max_): LLOQ: ± 20 %, other concentration levels : ± 15 % [ICH M10].

Table S6. Values of the correlations between random-effects estimated thanks to a variance-covariance matrix.

| **Correlations** | **Median [2.5%-97.5%]** |
| --- | --- |
| CL_TMP - CL_SDZ | 0.27 [-0.051 – 0.6] |
| V1_SDMX – CL_SDZ | 0.6 [-0.025 – 0.82] |
| V1_TMP – CL_SDZ | 0.5 [-0.18 – 0.79] |
| V2_SDMX – CL_SDZ | -0.54 [-0.79 – 0.032] |
| V2_SDZ – CL_SDZ | 0.87 [0.76 – 0.92] |
| V2_TMP – CL_SDZ | 0.19 [-0.073 – 0.58] |
| V1_SDMX – CL_TMP | 0.39 [-0.27 – 0.67] |
| V1_TMP – CL_TMP | 0.76 [0.57 – 0.87] |
| V2_SDMX – CL_ TMP | -0.62 [-0.91 - -0.082] |
| V2_SDZ – CL_TMP | 0.16 [-0.21 – 0.48] |
| V2_TMP – CL_TMP | 0.23 [-0.096 – 0.51] |
| V1_TMP – V1_SDMX | 0.65 [0.047 – 0.81] |
| V2_SDMX – V1_SDMX | -0.69 [-0.82 – -0.037] |
| V2_SDZ – V_SDMX | 0.59 [0.12 – 0.82] |
| V2_TMP – V1_SDMX | 0.29 [-0.11 – 0.69] |
| V2_SDMX – V1_TMP | -0.68 [-0.84 – -0.15] |
| V2_SDZ – V1_TMP | 0.48 [-0.14 – 0.75] |
| V2_TMP – V1_TMP | 0.29 [-0.18 – 0.66] |
| V2_SDZ – V2_SDMX | -0.46 [-0.73 – 0.011] |
| V2_TMP – V2_SDMX | -0.16 [-0.66 – 0.29] |
| V2_TMP – V2_SDZ | 0.42 [0.13 – 0.72] |

Abbreviations: CL = clearance; V1 = volume of the central compartment; V2 = volume of the peripheral compartment; SMX = sulfamethoxazole; SDZ = sulfadiazine; TMP = trimethoprim
